# Book Notices

**Published:** 1880-12-20

**Authors:** 


### *BOOK NOTICES.*

DISEASES OF THE PHARYNX, LARYNX AND TRACHEA. By Morrell Mackenzie, London, Senior Physician to the Hospital for Diseases of the Throat, at the London Hospital Medical College, and Corresponding Member of the Imperial Royal Society of Physicians of Vienna. New York: William Wood & Co., 27 Great Jones street.

A valuable illustrated work, of 418 octavo pages. The author states that "The work is based partly on the course of lectures which I have annually delivered at the London Hospital Medical College during the last twelve years, and partly on my essay on 'Diseases of the Larynx,' to which the Jacksonian Prize was awarded by the Royal College of Surgeons of England. Some of my lectures have appeared in the Journals, but by far the larger portion of the matter contained in these pages is now published for the first time."

His views on the treatment of syphilis he sumarizes as follows: (1) "That specific anti-syphilitic treatment is only required when serious constitutional symptoms are present; (2) that specific treatment in the early stages does not ward off the later manifestations of the affection; (3) that local treatment, analeptic remedies, and hygienic measures are of the utmost importance; (4) that the disease itself, except under unfavorable circumstances, tends toward spontaneous cure; and (5) that the development of serious pathological changes depends on conditions inherent in the patient himself.

It will, I hope, be understood that whilst employing iodide of potassium more frequently, I nevertheless consider mercury a valuable, and in some cases, an indispensible remedy."

### ANNUAL REPORT OF THE SURGEON GENERAL, UNITED STATES ARMY, 1880.

Surgeon General Barnes' Report for the fiscal year ending June the 30th, 1880, is a document of interest, evincing the characteristic ability and energy of this officer.

To the seven thousand eight hundred and twenty-eight (7,828) cases of injuries and operations reported in the Army of the United States, from the date of the publication of Circular 3, in 1871, to the close of the fiscal year ending June 30, 1879, have been added during the past year, one thousand and thirty-four (1,034) cases, making a total of eight thousand eight hundred and sixty-two (8,862) cases, viz: two thousand four hundred and ninety-nine (2,499) injuries of the head, one hundred and forty-one (141) of the face, sixty-five (65) of the neck, six hundred and sixteen )616) of the trunk, one thousand five hundred and seventy-six (1,576) of the upper extremities, one thousand and fifty (1,050) of the lower extremities; two thousand and fourteen (2,014) simple fractures, luxations and sprains, and nine hundred and one (901) injuries of a miscellaneous nature.

*Surgical Statistics of the War.*—Through correspondence with medical officers of the civil war, from reports of pension examiners, and from surgical journals and publications, additional data were obtained in three thousand eight hundred and eight (3,808) cases of injuries. Searches among the records of the Pension Office and of the Record and Pension Division of this Office gave further information in one thousand seven hundred and twenty-seven (1,727) and two thousand and eightytone (2,081) cases respectively.

### A PRACTICAL TREATISE ON SURGICAL DIAGNOSIS DESIGNED AS A MANUAL FOR PRACTITIONERS AND STUDENTS.

By Ambrose L. Ranney, A. M., M. D., Adjunct Professor of Anatomy and late Lecturer on the Surgical Diseases of the Genito-urinary Organs, and on Minor Surgery in the Medical Department of the University of New York; late Surgeon to the Northwestern and Northern Dispensaries, Resident Fellow of the New York Academy of Medicine, Member of the Medical Society of the County of New York, etc. Second edition—enlarged and revised. New York: William Wood & Co., 27 Great Jones street. W. B. Dalston, Agent, Atlanta, Georgia.

The work contains 471 large octavo pages, in cloth, beautifully printed in large, plain type. The second edition following so soon after the first, gives evidence of the demand for the work and its usefulness to the profession. The remarks in the Preface that he has followed the suggestions which have from time to time been made to him by instructors throughout the country, and endeavored to make the volumes especially valuable and attractive to the student in Medicine, as well as to those more advanced in the knowledge of the profession. The present volume is more full and complete than the first, and is in all respects a most practical and valuable work.

DANGERS AND TREATMENT OF EAR DISEASES. By Albert H. Buck, M. D., Aural Surgeon to the New York Eye and Ear Infirmary; Instructor in Otology, in the College of Physicians and Surgeons, in the City of New York. New York: William Wood & Co., 27 Great Jones street, 1880.

The above is an illustrated work of 400 octavo pages, in which the diseases of the Ear are presented in a text book form, and drawn from private and hospital practice; the treatment suggested having been found by experience to be safe and efficient. The work is full and practical, and constitutes a valuable addition to our works in this department.

COPY OF LINDSAY & BLAKISTON'S VISITING LIST FOR 1881. Thirteenth year of its publication. Always welcomed by the profession; Containing Almanac, Table of Signs, Poisons and Antidotes; The Metric System, and other information valuable to the Practitioner, at prices varying from $1.00 to $3.00, according to size, etc Philadelphia: Lindsay & Blakiston.

